# Stepwise approach for implementation of antimicrobial resistance surveillance in Africa

**DOI:** 10.4102/ajlm.v5i3.482

**Published:** 2016-10-31

**Authors:** Olga Perovic, Constance Schultsz

**Affiliations:** 1 National Institute for Communicable Diseases, National Health Laboratory Service and Department of Clinical Microbiology, University of Witwatersrand, Johannesburg, South Africa; 2 Department of Global Health, Amsterdam Institute for Global Health and Development, Academic Medical Center, University of Amsterdam, Amsterdam, the Netherlands

## Abstract

**Background:**

Antimicrobial resistance (AMR) has reached an end point, prompting a worldwide scare as no new antibiotics are in the pipeline, particularly for treatment of Gram-negative bacteria. To prevent further development and spread of AMR and to inform empirical treatment guidelines, surveillance of AMR is necessary.

**Objective:**

We aim to provide a framework for a stepwise approach toward implementation of laboratory-based surveillance for AMR in African countries.

**Methods and Results:**

Building up a surveillance system is a robust process that begins with a gap analysis in each participating country. This framework provides practical guidance on how to set up surveillance, identify responsibilities and set timelines in sustainable manner for African countries. It addresses sampling strategies, human resources, procurement and maintenance issues for AMR testing at routine clinical and national reference and public health laboratories involved in AMR surveillance. Key issues such as laboratory capacity building, training and continuous education, quality and diagnostic stewardship are discussed in detail.

**Discussion:**

There are several priorities for AMR surveillance that need to be addressed in a comprehensive manner at regional and national levels, whilst keeping in line with current and proposed initiatives for laboratory capacity building, in order for African countries to achieve goals for combatting the real and current threat of AMR.

## Introduction

Antimicrobial resistance (AMR) to bacterial pathogens is a global threat that ignores borders between countries. The emergence of multidrug-resistant bacteria means that very few effective antibiotics are available for the empirical or targeted treatment of many infectious diseases,^1,2^ including severe diseases such as bacterial bloodstream infections, bacterial meningitis and gonorrhoea. The impact that AMR and its current pace of expansion will have on economic and health outcomes cannot be underestimated.^3^ AMR is now considered a threat to global health security and, as such, is a key element of the Global Health Security Agenda (GHSA).^4^ The control of AMR requires a holistic approach involving human, animal and environmental domains. The strategies necessary to deal with the AMR threat developed by the World Health Organization (WHO) include: (1) to create awareness of AMR; (2) to introduce antimicrobial stewardship and appropriate use of antibiotics; (3) to restrict and regulate overuse of antibiotics in human and animals; (4) to improve infection prevention and control measures; and (5) to enhance surveillance of AMR.^5^ For some countries, AMR is already a high priority, whereas other countries are still in the initial stages of recognising it as a threat.^6^ However, we have now reached the point at which all countries, including low- and middle-income countries (LMIC), are required to develop strategies for containment of AMR as part of the WHO’s Global Action Plan (GAP) for AMR.^5^ This plan contains a number of features aimed at avoiding the emergence of new resistance and preventing transmission of existing resistance (Table 1).^7^

**TABLE 1:** Strategic planning for antimicrobial resistance interventions aligned with the World Health Organization Global Action Plan.^10^

Characteristics of strategies	National interventions
**General strategies applicable to containment of both emergence and transmission of resistance**	
Surveillance for AMR	Requires accurate data collection strategies on antimicrobial susceptibility testing capacity and results at all levels, delineated in national policies and plans
Economic assessment of sustainable investment for AMR surveillance	Requires commitment at national and international level
**Strategies for containing emergence of resistance**	
Education of all professionals involved in dealing with antimicrobials	Requires commitment at local and national level
Education of patients and public on inappropriate use of antimicrobials	Requires commitment at national level
Rapid diagnosis of bacterial and viral infections	Requires infrastructure at facility levels
Provide guidelines for antimicrobial susceptibility testing and implementation of accreditation for performing laboratories	Requires national commitment at facility levels
Development of treatment guidelines to prevent inappropriate use of antimicrobial agents and development of policies to regulate prescription requirements, and other regulations	Requires national and facility involvement
Development of antimicrobial stewardship programmes	At national and facilities levels
Provide EPI programmes to all	National policy
**Strategies for containing transmission of resistance**	
Improve nutrition in order to reduce susceptibility to infections	National and local
Hand washing	National guidelines and facility levels implementation
Isolation of patients with emerging AMR pathogens	National guideline and facility levels implementation
Increase number of hospital beds	National level and facility levels implementation
In emergence of resistance screening for new cases	National level and facility levels implementation

AMR, antimicrobial resistance; EPI, expanded programme on immunisation.

Most information on AMR prevalence comes from high-income countries, and very little is known about AMR in African countries. A systematic review of AMR in sub-Saharan Africa by Leopold et al. revealed a high prevalence of AMR to commonly-used antibiotics in clinical bacterial isolates.^8^ That study also underlined the flaws in currently-available data and the challenges faced in LMICs when implementing AMR surveillance, including a lack of allocated human and economic resources.^8^

As one of its multifaceted responses to combat AMR, the first step proposed in the WHO GAP is to perform a baseline assessment of the AMR prevalence in all countries.^9^ The WHO has sent an open call to all countries to enroll in the Global AMR Surveillance System and to participate in a structured surveillance programme.^6^ LMICs are encouraged to use every opportunity to apply for transfer of resources and funds in order to comply with the GAP. There are clear and sometimes large differences between countries in the African region as to what extent AMR surveillance is currently taking place. These differences are dependent on many factors, including socio-economic factors; however, surveillance of AMR is most likely to be successfully implemented when approached using a stepwise process and when tailored to the country’s level of preparedness.^10^ Here we address the essential components of an AMR surveillance implementation plan.

In general, an AMR strategy is a holistic approach that includes monitoring resistance trends, strengthening diagnostic laboratories, applying infection prevention and control measures to increase social awareness and international collaboration, strengthening drug regulation and supply chains and development of novel drugs. All of these factors are aimed at promoting antimicrobial stewardship and encouraging novel research in diagnostics and drug development. AMR surveillance is an important process for estimating the extent of the resistance burden in each country. AMR surveillance relies on diagnostic laboratories; in sub-Saharan Africa, the need for laboratory improvement is evident, with some countries in need of laboratory system built from the ground up. This prevalent lack of laboratory resources and the subsequent difficulty of obtaining accurate results on antimicrobial susceptibility testing is an important challenge to address when proposing a stepwise approach. In addition, an AMR strategy should consider surveillance for resistance of antibiotics used in animal feed and structure the strategy the same way as for humans. The WHO GAP emphasises the One Health approach,^5^ which is a multi-sectorial and multi-disciplinary way to optimise use of antimicrobials among both humans and animals. Monitoring and evaluation of all tasks should be implemented with clear indicators and targets within the AMR strategic framework. Finally, the benefits of AMR surveillance for clinically-relevant reporting should be made apparent (Table 2).

**TABLE 2:** Benefits of antimicrobial resistance surveillance.

AMR surveillance benefits	Action taken
Evidence based public health policy	Changing public health policy such as notifying MDRO
Monitoring trends in AMR	Based on increase or decrease in trends of resistance recommendation is issued
Public health intervention	Introduction of pneumococcal conjugate vaccine in EPI
Treatment guidelines	Continuous updates based on AMR surveillance
Continuous education and training	Integrated in antimicrobial stewardship programmes
Epidemiological studies and research	Based on strain phylogeny pathogen emerging and outbreak detection

AMR, antimicrobial resistance; MDRO, multidrug-resistant organism; EPI, expanded programme on immunisation.

## Surveillance objectives

The key objectives of AMR surveillance are to estimate trends in AMR rates and to detect the emergence and potential spread of AMR, in order to inform programmes that formulate guidelines for treatment and prevention of infections and prevention of AMR transmission. In Africa, the development and improvement of laboratory capacity, including standardised testing, external quality assessment programmes, procurement, and timely and cost effective reports, should eventually lead to the establishment of an integrated and coordinated surveillance system for AMR in the region, which will strengthen knowledge about AMR through surveillance and research. This surveillance should increase understanding of the implications of AMR and its epidemiology and allow for monitoring the effectiveness of guidelines and policy implementation. In addition, it would permit research and development of new diagnostics and novel technologies, optimisation of treatment, and other interventions as well as build human resource capacity as a secondary objective. Collaboration within a team of human and animal experts in AMR surveillance would allow for a One Health approach. This requires the establishment of coordinated surveillance in animals, the ability to control and regulate antibiotics use in animals, and the identification of alternative options for growth promoters in agriculture.

## Antimicrobial resistance surveillance approaches

There are several approaches toward surveillance of AMR, including population-, sentinel- and laboratory-based surveillance. The general perception is that laboratory-based surveillance is currently the most efficient method of surveillance of AMR, which is what the WHO and GHSA advocate.^1,4,5^ However, laboratory-based surveillance is often biased, because of the potential barriers to and selection processes for submission of clinical specimens to laboratories for culture and susceptibility testing, particularly in resource-constrained settings.^2^ This bias may result in laboratory-based surveillance data being skewed toward a higher prevalence of AMR. Therefore, countries may choose to perform targeted population-based surveillance for specific diseases or pathogens, where there is a clear need to follow AMR trends over time. Given the current recommendations from the WHO and GHSA, the focus of this paper will be on laboratory-based surveillance.

**FIGURE 1:**
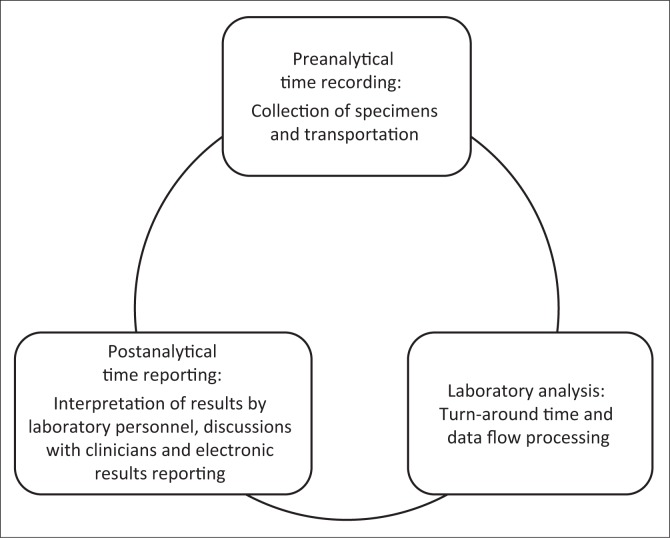
Laboratory processes in relationship to electronic collection of antimicrobial resistance data.

For laboratory-based surveillance to yield comprehensive data, a functional infectious disease diagnostic cycle is required. This cycle includes clinicians submitting samples for culture and susceptibility testing, a bacteriology laboratory that can generate quality culture and susceptibility test results, and a reporting system that includes not only the clinician requesting the test, but also a laboratory information system (LIS) that can inform the surveillance programme, which may be steered by a central body (Figure 1). In addition, for laboratory surveillance programmes that are technically carried out by national public health laboratories, a crucial element of the cycle is the rapid and complete transfer of all required materials, isolates and/or data from peripheral laboratories to the national site, and back reporting of results to ensure continuous engagement of laboratories and clinicians.

**FIGURE 2:**
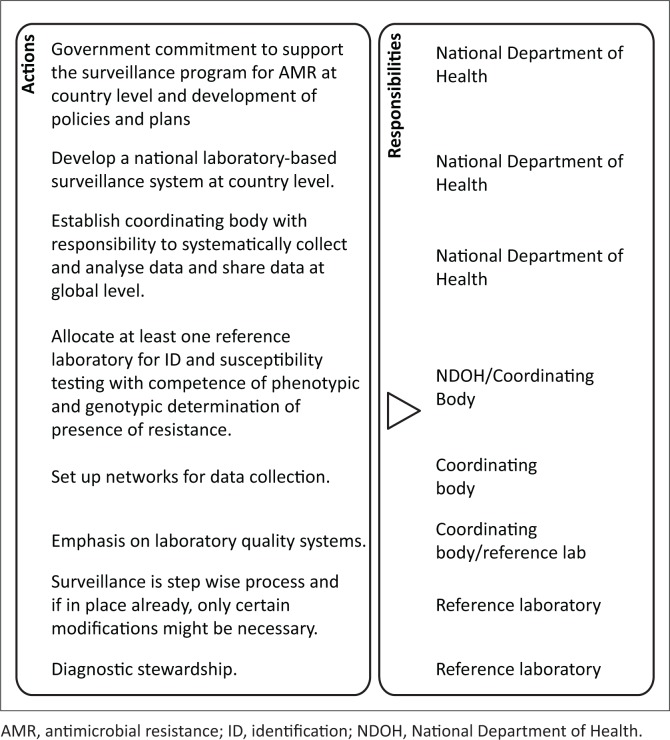
Surveillance programme summary.

For a national laboratory-based surveillance programme to be successful, government commitment to support the surveillance programme for AMR at the country level is essential (Figure 2). Such commitment can best be demonstrated by the design of dedicated policies and plans for AMR control at government level, which include implementation plans and budgets. Governments are required to provide human and financial resources, which may come from a variety of sources. Governments of some countries may need to address specific deficiencies in supplies of water, electricity and infrastructure to allow laboratories to function appropriately. The national AMR surveillance implementation plan could be used as an advocacy tool for government funding to achieve this. Departments of Health and other governmental structures should work in conjunction with partners, such as the WHO, GHSA and the African Society for Laboratory Medicine (ASLM), on AMR programmes that require an inter-sectorial approach. The roles of WHO and ASLM should be in capacity building, financial support, promoting and communicating abroad on the AMR framework.

Establishment of a national laboratory-based surveillance system at the country level, as established by each country’s National Department or Ministry of Health, also includes the establishment of a coordinating centre with the responsibility to systematically collect, analyse and share data at the national and international level. Governments may decide to set up their coordinating centres differently, but all will need expertise in epidemiology, clinical microbiology, infectious diseases, veterinary medicine, data management and governance. Such expertise may be built and subsequently sustained through continuous education programmes that take place locally or within the region. For example, South Africa, Kenya, Ghana, Uganda, Nigeria and Burkino Faso all provide a Field Epidemiology Training Programme.

Establishment of at least one reference laboratory that can undertake infectious disease diagnostics and antimicrobial susceptibility testing, and that has competence in phenotypic and genotypic confirmation of the presence of resistance genes, is required. Initially, in the absence of capacity to perform advanced phenotypical and molecular testing in the national reference laboratory, a clinical hospital bacteriology laboratory with demonstrated capacity to perform these functions, could be selected as a reference laboratory. In addition, instead of performing genotyping at the national level, molecular typing and gene characterisation could take place in another reference laboratory in the African region. National reference laboratories perform their role in close collaboration with provincial and district clinical laboratories, which generally receive samples directly from patients. Together, these laboratories form the national surveillance network. Countries should also consider including private laboratories in their network. Although specifics vary by country, such laboratories may receive large numbers of samples from a substantial proportion of the population. Strategies on how to involve and include private laboratories in national AMR surveillance networks must be developed to achieve this inclusion.

Setting up a regional network for AMR surveillance in Africa to provide locally relevant AMR data to serve as a bench mark may be the eventual goal. Whilst several regional networks exist, the Central Asia and Eastern European Surveillance of Antimicrobial Resistance network is one model for the African region. This network is a joint initiative of the WHO Regional Office for Europe, the European Society of Clinical Microbiology and Infectious Diseases, and the Dutch National Institute for Public Health and the Environment. The Central Asia and Eastern European Surveillance of Antimicrobial Resistance network includes all countries in the WHO European Region that are not part of the European Antimicrobial Resistance Surveillance Network, which is coordinated by the European Centre for Disease Prevention and Control in the European Union.^11^ The organisation of regional AMR surveillance programmes creates opportunities for concentrating specific expertise in dedicated national laboratories, thus potentially reducing costs and increasing efficiency. In addition, such regional programmes may provide support for solving logistical issues, such as those described below.

## How to enable microbiology laboratories to introduce antimicrobial resistance surveillance

Laboratories that are participating in AMR surveillance as sentinel or reference sites should comply with baseline requirements as indicated under this heading. The quality of laboratory-based AMR surveillance data depends to a large extent on the microbiology laboratory. Firstly, the quality of a laboratory system should be assessed in order to identify gaps and areas for improvements. In order for participating laboratories to meet minimal quality standards requirement as defined by the network (e.g., accreditation, external quality assessment programme, and other standards), establishment of a laboratory quality system is essential. However, the logistical difficulties of joining external quality assessment programmes and receiving quality control materials present significant challenges to countries in sub-Saharan Africa, because of customs and other policies for shipment of samples and microorganisms between different countries.

Laboratory assessments should include pre-analytical, analytical and post analytical steps for reporting of antimicrobial susceptibility testing (AST) and its limitations. For the pre-analytical phase, diagnostic stewardship is critically important. Diagnostic stewardship is the process that guides and improves the use of microbiological tests to ensure they are timely, appropriate and accurate. Reports sent to clinicians should include a feedback form to help on-going improvements. To apply required standards for AST, laboratories must have the required infrastructure, equipment, supplies and staffing in place. Laboratories should be able to identify gaps and recommend improvements. For example, some laboratories may have all the equipment needed but no supply of reagents or other consumables needed for basic microscopy, culture and AST implementation. This, in turn, affects diagnostic stewardship as clinicians may lose confidence in laboratory reporting when timely, appropriate and accurate results cannot be achieved. A country’s national AMR surveillance plan and the regional AMR surveillance network may provide for policies and procedures that make timely ordering and receipt of consumables and other supplies feasible and affordable. ASLM could play an active role in such advocacy activities. Laboratory experts should be involved in ordering supplies to avoid receiving supplies that are irrelevant, not useful or of poor quality.

The use of a LIS is limited in most LMICs, which is unfortunate, as it improves AMR surveillance by reducing workload and errors, thus improving the overall quality of microbiology laboratories. Laboratories should use LIS to retrieve and share AST data with national and regional bodies (Figure 1). Whilst databases preferably allow sharing of data, confidentiality must also be guaranteed. The WHONET data management software provides these features and is freely available.^12^ Other initiatives such as those from the RESAOLAB network^13^ have created their own open source LIS that includes an option to store and report AMR data.

As mentioned, national and regional networks will require (inter)national standardisation of methods. For standardisation of bacteriology test methods, guidelines are available and are often used, e.g. those from the Clinical and Laboratory Standards Institute^14^ or from EUCAST,^15^ the latter of which is freely available. For additional standardisation of data collection and reporting to a central data collection point, a regional network would require additional software that can be linked to local LISs, including newly-designed or WHONET, European Antimicrobial Resistance Surveillance Network, or Central Asia and Eastern European Surveillance of Antimicrobial Resistance network software. This software may be expensive to set up, and maintenance is an additional consideration. It is also important to consider that these software systems may not be sufficient for outbreak detection due to the lack of an alert system. In addition, such systems must be compatible with existing or future national LIS systems, in order to prevent time-consuming transfers of data, as is currently needed for WHONET. Thus, the available data collection systems for each country and the costing structure for implementation of region-wide AMR surveillance need further investigation.

Human resource capacity may be the most challenging issue, as shortages of qualified and trained professionals can have a serious negative impact on the ability of laboratories to perform testing. Human resources are a weak spot in microbiology and have been ignored. Quality microbiology requires sufficient number of qualified technicians, as well as supervision by MD or MSc level clinical microbiologists who are capable of reporting results to clinicians. Finally, veterinarians should be involved in the process of developing surveillance systems to strengthen diagnostic stewardship in animal health, provide assistance about status of AMR in animal health, and implement preventative measures in animal health, if needed at a later stage. For capacity building that includes education, training and refresher courses and other forms of networking, the WHO and ASLM should be excellent sources of support.

## Implementation of laboratory-based antimicrobial resistance surveillance

The following components should be considered when designing an AMR surveillance plan (Table 3).

**TABLE 3:** Design summary for development of laboratory-based surveillance matrix.

Designations†	Target milestones†	Responsibilities
Establishment of a Coordinating Centre (CC)	Composition and development of terms of reference Contact details of the CC shared with stakeholders	At national level
Acceptance of the generic laboratory-based surveillance document on AMR WHO GLASS	Technical meeting for adoption of the key elements of the guide. This will allow staff involved in AMR surveillance to learn and comply with its objectives and content. (CLSI or EUCAST guidelines are subject to change annually).	National Coordinating Centre
Designation of the national reference laboratory for AMR at country level	Selection of one national reference bacteriology laboratory with capacity for AST for bacterial organisms selected for surveillance in phased approach. Define or update the terms of reference of the national reference laboratory related to AMR surveillance	Technical support to develop an appropriate base line level for surveillance
Gap analysis Assessment of the capacity for AMR surveillance, identification of the gaps and development at local and national levels	Assessment of laboratory capacity using existing ISO standardised tools and proposed key activities needed to be supported, based on findings of the assessment and the requirements of the national guidelines on AMR	National Coordinating Centre
Implementation of laboratory-based surveillance for AMR	Key action points: Identification of surveillance sites Creation of a laboratory and surveillance national network Updating or creating new SOPs for the network Procuring essential reagents and supplies for the national reference laboratory and for the surveillance sites Ensuring the laboratories have adequate personnel and (on-going) training of staff Active participation in bacteriology PTS Collection and sharing of data on AMR Monitoring, evaluating and improving the implementation process Providing regular annual reports on AMR to local, national and regional reporting structures including WHO Involvement of private laboratories in the network	National Reference Laboratory

† Activities are presented in a stepwise approach starting with the first of a series of steps.

AMR, antimicrobial resistance; WHO, World Health Organization; GLASS, Global AMR Surveillance System; CLSI, Clinical and Laboratory Standards Institute; AST, antimicrobial susceptibility testing; ISO, International Organization for Standardization; SOP, standard operating procedure; PTS, proficiency testing schemes.

### Gap analysis

A gap analysis that includes all levels of the health system must be performed in order to implement or further expand existing AMR surveillance activities. For example, it was recently reported that many countries still lack reference to laboratories in their health policies and plans and do not consider laboratories at all in their health budgets.^16^ An analysis of existing surveillance systems should be undertaken before development of a new system is considered.

There are currently several tools available to assess laboratory capacity and quality that could be used as a part of a gap analysis. These include the Stepwise Laboratory Improvement Process Towards Accreditation tools developed by WHO,^17^ as well as by ASLM as part of the GHSA.^18^ These tools are targeted at different levels of the health system. The WHO Stepwise Laboratory Improvement Process Towards Accreditation tool is a generic quality system assessment tool for laboratories and is not designed to look specifically at AMR surveillance capacity.^19^ The audit checklist tools developed by ASLM are established to assess laboratory capacity at national level and include a bacterial AMR testing module. Whichever tool is used, it should be applied for the purpose for which it was designed.

### Priority pathogen-antibiotic combinations

In order to ensure AMR surveillance of relevant pathogens, it is important to establish case definitions. For example, standard definitions should be established for patients with symptoms of syndromes that have high clinical importance, such as central nervous system infections and septicaemia. The selection of specimens should be directed according to local, national, and regional relevance, after considering site of infection, such as meningitis or bacteraemia. Isolates from cerebrospinal fluid and blood cultures, with particular pathogens of focus, including both Gram-negative and Gram-positive bacterial organisms, should be included. In addition, exclusion criteria, such as de-duplication based on one specimen per patient in 21 days or based on Global AMR Surveillance System criteria,^6^ should be established.

In addition, there are number of priority organisms and antibiotics recommended by WHO that should be considered in a stepwise process. These include *Enterococcus faecium, Staphylococcus aureus, Klebsiella pneumoniae, Acinetobacter baumannii, Pseudomonas aeruginosa, Enterobacter* spp., *Streptococcus pneumoniae*, *Salmonella* spp., *Shigella* spp. and *Neisseria gonorrhoea*. Each country should prioritise which drug/bacteria combination to test and report according to local relevance.

### Surveillance methods

**FIGURE 3:**
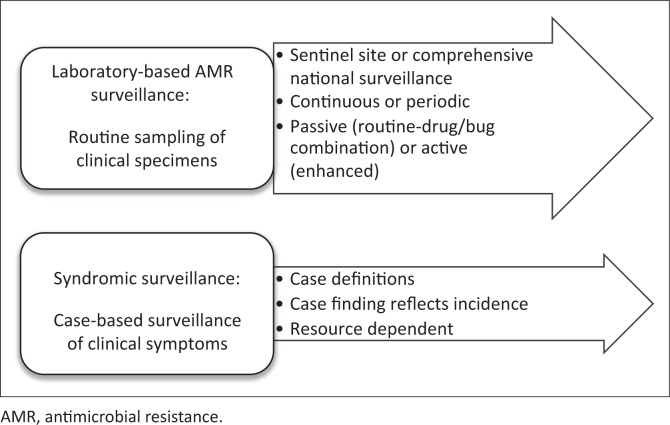
Surveillance methods.

There are several types of surveillance and current recommendations are to perform laboratory-based surveillance. For each drug/bacteria combination, the approach toward laboratory-based surveillance should be decided on (Figure 3). Whichever approach is chosen, the availability of basic microbiology and core patient data is imperative and methods to calculate aggregate data based on sampling methods (periodic or continuous) are required. Each country should decide how to approach the surveillance methodology that is sustainable in the defined period of time available.

### African regional antimicrobial resistance reference laboratory

For technical support and strategy advice, an AMR reference laboratory for African region should be established. The role of an African region AMR reference laboratory would be multi-faceted. Firstly, the regional reference laboratory should provide training of laboratory personnel from network member countries to help build capacity for development of surveillance systems in the trainees’ home countries. Secondly, the regional reference laboratory should take the lead in introducing and providing training on new techniques. Thirdly, it should provide confirmation of difficult AST results and offer advice when necessary. Finally, it should manage other laboratory networks in the region. A public/private partnership should be sought as the funding source.

## Discussion and conclusion

Considering the growing impact of AMR worldwide and the lack of quality surveillance systems in the African region, an enormous effort must be undertaken through international and multi-sectorial collaboration. A recent report analysing the AMR surveillance capacity of the East Africa Public Health Laboratory Network by the Center for Disease Dynamics, Economics & Policy, commissioned by the World Bank, summarised the following key issues for laboratories to carry out AMR surveillance:^20^

- Lack of demand of bacteriology diagnostics from clinicians, related to length of time to get results (at least two days); lack of trust in results; lack of laboratory capacity for blood cultures, which are needed for many of the most serious, life-threatening infections.
- Low priority given to bacteriology diagnostic supplies by hospitals and other decision makers who control purchasing.
- The multi-component nature of bacterial culture and antimicrobial susceptibility testing, which makes it especially vulnerable to weak supply chains and frequent stock outs; results cannot be obtained if essential components are unavailable when testing is needed.
- Lack of recognition that microbiology requires dedicated, trained personnel, leading some facilities and/or ministries of health to rotate staff in and out of microbiology.
- Few options for automated testing relative to haematology, chemical pathology or HIV testing, which may be more satisfying to staff, leading to low morale.
- Requirement for patients to pay out of pocket for diagnostic tests in many countries.

Whilst some of these may be generic to LMICs, others may be more specific to certain countries or settings.^21,22^ Whichever the situation, solving these issues will require collaborative efforts of multiple stakeholders. ASLM could play a key role in facilitating these efforts due to its central position between ministries of health, policy makers, laboratory professionals and, increasingly, clinicians and professional societies.

In conclusion several key steps should be implemented for an AMR surveillance programme such as: assessment of laboratory capacity to perform surveillance for AMR; identification of gaps; adoption of the Global AMR Surveillance System manual or other guidelines; designation of national reference laboratories for AMR and organisation of advisory committees; implementation of laboratory-based AMR surveillance; and reporting to the Global AMR Surveillance System.
